# Somatic mutation detection and *KRAS* amplification in testicular germ cell tumors

**DOI:** 10.3389/fonc.2023.1133363

**Published:** 2023-03-16

**Authors:** Eduardo R. M. Cabral, Marilia F. Pacanhella, Andre V. H. Lengert, Mariana B. dos Reis, Leticia F. Leal, Marcos A. de Lima, Aline L. V. da Silva, Icaro A. Pinto, Rui M. Reis, Mariana T. Pinto, Flavio M. Cárcano

**Affiliations:** ^1^Molecular Oncology Research Center, Barretos Cancer Hospital, Barretos, Brazil; ^2^Barretos School of Health Sciences Dr. Paulo Prata – FACISB, Barretos, Brazil; ^3^Life and Health Sciences Research Institute (ICVS), Medical School, University of Minho, Braga, Portugal; ^4^3ICVS/3B’s-PT Government Associate Laboratory, Braga, Portugal; ^5^Division of Genitourinary Medical Oncology, Oncoclínicas, Belo Horizonte, Brazil

**Keywords:** testicular neoplasms, mutation, testicular germ cell tumor, K-ras Gene, TP53 Gene, next-generation sequencing

## Abstract

**Background:**

Testicular Germ Cell Tumors (TGCT) are the most common cancer among young adult men. The TGCT histopathology is diverse, and the frequency of genomic alterations, along with their prognostic role, remains largely unexplored. Herein, we evaluate the mutation profile of a 15-driver gene panel and copy number variation of *KRAS* in a large series of TGCT from a single reference cancer center.

**Materials and methods:**

A cohort of 97 patients with TGCT, diagnosed at the Barretos Cancer Hospital, was evaluated. Real-time PCR was used to assess copy number variation (CNV) of the *KRAS* gene in 51 cases, and the mutation analysis was performed using the TruSight Tumor 15 (Illumina) panel (TST15) in 65 patients. Univariate analysis was used to compare sample categories in relation to mutational frequencies. Survival analysis was conducted by the Kaplan–Meier method and log-rank test.

**Results:**

*KRAS* copy number gain was a very frequent event (80.4%) in TGCT and presented a worse prognosis compared with the group with no *KRAS* copy gain (10y-OS, 90% *vs*. 81.5%, p = 0.048). Among the 65 TGCT cases, different variants were identified in 11 of 15 genes of the panel, and the *TP53* gene was the most recurrently mutated driver gene (27.7%). Variants were also detected in genes such as *KIT*, *KRAS, PDGFRA*, *EGFR*, *BRAF*, *RET*, *NRAS*, *PIK3CA*, *MET*, and *ERBB2*, with some of them potentially targetable.

**Conclusion:**

Although larger studies incorporating collaborative networks may shed the light on the molecular landscape of TGCT, our findings unveal the potential of actionable variants in clinical management for applying targeted therapies.

## 1 Introduction

Testicular germ cell tumors (TGCTs) are relatively rare tumors, accounting for about 1.7% of all cancers (1). However, they are the most common malignant tumors among men aged between 15 and 44 years worldwide (1). TGCTs are highly curable, and since the employment of platinum-based combination chemotherapy regimens began (2, 3), the overall cure rates have dramatically increased to over 95% across all disease stages, however cure rates for IGCCCG poor risk disease remain less than 80% (4–8). Ten to twenty per cent of patients with advanced disease are resistant to cisplatin and present with a less favorable prognosis, with relapses requiring second-line treatment (9, 10).

The process of gonocyte maturation and cell differentiation driven by mutations, associated with the microenvironment interaction, leads to the formation of *in situ* germ cell neoplasms (GCNIS) (11, 12). These precursor cells remain senescent until puberty and develop into TGCT under the effect of sex hormones and additional genetic changes (11). The TGCTs are subclassified into two major histological groups: seminomas and non-seminomas. Seminomas are formed by cells similar to GCNIS, with suppressed differentiation, high sensitivity to chemotherapy and radiotherapy, and better prognosis, whereas non-seminomas have different histologies due to greater differentiation (embryonic carcinoma, teratoma, yolk sac tumors, and choriocarcinoma), resistance to radiotherapy and more aggressive behavior (13).

The genetic composition of TGCTs portrays the embryonic characteristics of their precursor, the primordial germ cells (PGCs). The molecular biology of TGCTs is not still fully elucidated, despite intensive research on the molecular mechanisms involved in their development (14, 15). Copy number alterations are the most consistent genomic alterations in all histological subtypes. Aneuploidy is considered an important biomarker for TGCT, with the number of chromosomes varying between 50 and 70, and gains in the short arm of chromosome 12 (isochromosome 12p) (16–20).

Recurrent somatic mutations in TGCTs are low, with the exception of variants in *KIT* and *KRAS* oncogenes that are frequently described (21, 22). *KIT* and *KRAS* mutations are reported more frequently in seminomas when compared with non-seminomas (23–25). Moreover, high levels of expression and *KRAS* gene amplification have been described as mutually exclusive events in TGCTs (26, 27). The molecular profile of Brazilian TGCT is poorly explored, and the mutational landscape remains unknown (15, 28–30).

Importantly, a molecular understanding of TGCTs might unveil signatures that will guide a more specific and effective treatment, as well as medical decision-making. Therefore, the present study aims to evaluate the mutational profile of driver genes and *KRAS* copy number variation in Brazilian TGCT patients.

## 2 Materials and methods

### 2.1 Study population material

We performed a retrospective cohort study of all TGCT Brazilian patients who attended Barretos Cancer Hospital. This study was approved by the Barretos Cancer Hospital IRB under Protocol No. 784/2014. Clinicopathologic data were retrieved from the electronic medical records, including data on age, date of initial diagnosis, histological type, pathologic diagnosis, overall survival, and treatment history with chemotherapy. All information that could identify the patients was collected and managed using REDCap electronic data capture tools hosted at Barretos Cancer Hospital (31, 32), to ensure the confidentiality of the data and the anonymity of the patients. REDCap (Research Electronic Data Capture) is a secure, web-based software platform designed to support data capture for research studies, providing 1) an intuitive interface for validated data capture; 2) audit trails for tracking data manipulation and export procedures; 3) automated export procedures for seamless data downloads to common statistical packages; and 4) procedures for data integration and interoperability with external sources.

Formalin-fixed paraffin-embedded (FFPE) tumoral tissues were retrieved by macrodissection guided by hematoxylin and eosin-stained slides. The macrodissection of mixed tumor cases was performed combining all histology subtypes in the sample. All the samples are from primary tumor before any systemic treatment exposure and were reviewed by an independent pathologist. Biological samples of fresh frozen tumoral tissue were retrieved at the Biobank of the Barretos Cancer Hospital (BB-BCH), ensuring the quality of processes and the suitability of the biospecimens for research (33).

For mutational analysis, among the 183 cases diagnosed with TCGT feasible for inclusion in this study, 75 did not meet the quality control criteria for DNA extraction (minimum 70% tumor; maximum 30% necrosis); so 108 cases had their genetic material extracted. After the multiplex PCR for the GAPDH gene, used as a quality control for FFPE samples, 12 cases did not present intact and good quality DNA and, for this reason, could not proceed to the preparation of libraries for sequencing. Of the 96 samples that went on to prepare libraries, 32 did not have their targets amplified. Ultimately, only 65 cases were included for the Next Generation Sequencing (NGS) variant identification analyses. For Copy Number Variation (CNV) analysis for KRAS, among the 183 cases diagnosed with TCGT and feasible for inclusion in this study, 66 cases did not have fresh frozen tissue samples available in the Biobank. Out of 117 cases with available samples, seven post-chemotherapy cases were excluded. Among the 110 cases feasible for inclusion in this study, 59 did not meet the quality control criteria for DNA extraction (minimum 70% tumor; maximum 30% necrosis). Ultimately, only 51 cases were included for the CNV analysis for KRAS gene. The cases selection flowchart is presented in the **Figure S1**.

### 2.2 DNA isolation and integration analysis

FFPE samples were kept in the stove for 20 min at 80°C. The rehydration of the sample was then performed by incubation with decreasing alcohol percentages (100, 70, and 50% ethanol) for 1 min at room temperature (RT). After that, samples were left in milli-Q H_2_O until the extraction step. Tumor DNA from FFPE was extracted using QIAamp DNA FFPE Tissue Kit (Qiagen), following the manufacturer’s protocols as previously reported (28). Tumor DNA samples were quantified using Qubit^®^ 2.0 Fluorometer technology (Invitrogen). A multiplex PCR for *GAPDH* gene was used as quality control for FFPE samples, and those with amplifications of longer fragments (200bp, 300bp, and 400bp) (34) were considered eligible for NGS workflow. Amplifications were performed with a final volume of 30 µL, containing 1.5 mM MgCl_2_ (Invitrogen), 0.2 mM dNTPs (Invitrogen), 0.133 µM of each primer, 1 unit of Taq DNA Polymerase (Invitrogen), and 50 ng of tumor DNA. The reactions were performed in a ProFlex thermocycler (Thermo Fisher Scientific) using the following amplification parameters: 94°C for 4 minutes, 35 cycles of 94°C for 1 minute, 56°C for 1 minute, 72°C for 1 minute, and a final extension of 72°C for 7 minutes. The amplified DNA was assessed on a 2% agarose gel and only samples with 200 bp or more were accepted for further preparation of sequencing libraries for mutation analysis.

Tumor DNA from fresh frozen tissue samples was extracted using the DNeasy Blood and Tissue kit (Qiagen^®^ Hilden-Germany), following the manufacturer’s instructions, and by Biobank Barretos procedures (NEUBER et al., 2021). DNA was quantified using NanoDrop™ Spectrophotometer (Thermo Scientific).

### 2.3 Copy number variation analysis of *KRAS* gene

For CNV analysis (n=51), a specific TaqMan assay for the *KRAS* gene (Hs02739788_cn—Thermo Fisher Scientific) was used. The reference gene Ribonuclease P (RNAse P 4403326 —Thermo Fisher Scientific), located on chromosome 14, is known to contain two copies in the human genome and was used to calculate the number of copies of the target gene. The TaqMan^®^ *KRAS* Copy Number Assay contained two primers and an FAM™ dye-labeled MGB probe to detect the target genomic DNA sequence; the Ribonuclease P reference assay contained two primers and a VIC^®^ and TAMRA™ dye-labeled probe to detect the genomic DNA reference sequence. As controls for *KRAS* copy number, DNA from the GCT cell line (NTERA-2), which contains five copies of *KRAS* and a normal blood sample, which contains two copies, were used. The qPCR reactions were performed using 20 ng of DNA, 10 μL TaqMan Genotyping Master Mix (Thermo Fisher Scientific), 1 μL of probes and region-specific primers and controls, and nuclease-free ultrapure water in a total volume of 20 μL. The copy number variation assays were performed simultaneously with the reference assay in a qPCR duplex using QuantStudio 6 equipment (Thermo Fisher Scientific) and cycling with an initial temperature of 95°C for 10 minutes, followed by 40 cycles of 95°C for 15 seconds, and 60°C for 1 minute. The Ct values of each reference sample and gene were exported to the CopyCaller™ software (Applied BioSystems, USA), in which a comparative Ct quantification (ΔΔCt) analysis of the real-time data was performed. The comparative Ct (ΔΔCt) method calculated the difference (ΔCt) between the threshold cycles of the target and reference assay sequences and then compared the ΔCt values of the test samples to a calibrator sample (normal blood), which contained a known number of copies of the target sequence (two copies). All qPCR reactions were performed in technical triplicates. A variation in the number of copies of the *KRAS* gene was considered when the cases presented a copy number different from two for this gene. *Via* the method used, it was not possible to differentiate gain from amplification, only to define a variation in the number of copies different from normal (two copies of the *KRAS* gene).

### 2.4 Mutational analysis

To evaluate the driver mutations (n=65), the TruSight Tumor 15 panel (TST15 - Illumina) was used, as reported by the manufacturer’s instructions (35). The panel provides a comprehensive assessment of 15 driver genes (*TP53, KRAS, KIT, NRAS, BRAF, EGFR, MET, PIK3CA, PDGFRA, AKT1, ERBB2, RET, GNA11, GNAQ*, and *FOXL2*). For library preparation, targets were enriched in a multiplex PCR, with an initial input of 20 ng/μL of DNA, according to the manufacturer’s instructions. After library preparation, the DNA amplification was verified by electrophoresis. The libraries were paired-end-sequenced using MiSeq Reagent Kit v3, 600 Cycles on the Illumina MiSeq instrument (Illumina). Demultiplexed data and FASTQ files were generated using BaseSpace software (Illumina).

The reads were aligned with the reference genome (GRCh37/hg19) using the algorithm BWA-MEM-PAIRED, and Sequence Alignment Map (SAM) output format files were converted to the Binary SAM (BAM) format using SAMtools. BAM files were processed using the Genome Analysis ToolKIT (GATK). Realignment and variant calling were performed using GATK HaplotypeCaller and the Freebayes genetic variant detector using BED–Capture. The annotation of variants was performed using the Varstation^®^ (36) analysis platform, *via* the Varstation Annotation Somatic software.

A customized analysis filter was used, which retained pathogenic and likely pathogenic variants of uncertain/unknown significance (VUS) and drug response, as well as those with population frequency less than 1% (gnomAD, 1000 genomes, ABRaOM); allelic frequency (VAF) greater than or equal to 3%; vertical coverage (depth) greater than or equal to 500x in the exonic and splicing regions, and frameshift, non-synonymous, or stopgain type. Variants leading to loss of function, “high‐impact” variants (frameshift, nonsense, and canonical splice site variants), and missense variants were selected. In addition, benign and likely benign variants were excluded from the analyses.

The manual curation of the variants was carried out using public databases (Clinvar, IARC *TP53*, Varsome, COSMIC, gnomAD, ABraOM, and CGI) for the better classification of the detected variants according to their effect, pathogenicity, and frequency in the general population. CGI was also employed to verify the status of driver variants, and only known variants or those predicted as drivers in cancer-related genes were retained. The variants were classified according to public databases as pathogenic, likely pathogenic, uncertain significance (VUS), and drug response.

### 2.5 Statistical analysis

Univariate analysis was used to compare sample categories in relation to mutation frequencies. For this, the χ2 test or Fisher’s exact test was used, according to the characteristics of the sample. Survival curves were plotted using the Kaplan–Meier method and events of interest (death record) were considered for the outcome of overall survival (OS). Alive patients and those lost to follow-up were discounted. Univariate comparisons of survival times were performed using the log-rank test. Statistical analyses were performed using IBM SPSS Statistics for Windows, Version 2.0 (IBM), and alpha=0.05 with 80% power in a two-tailed hypothesis test.

## 3 Results

### 3.1 Clinicopathologic characterization of TCGT patients

Out of the 97 cases analyzed, the average age at diagnosis was 32.5 years (range, 18-50 years), and most cases (57.7%) were non-seminomas (**Table 1**). Among the histological group of non-seminomatous tumors, 41.2% presented a histology of mixed tumors. In total, 67% of cases were in the advanced stages of the disease (stage II and III—AJCC), of which 39.2% were low risk, according to the IGCCCG risk.

**Table 1:** Characteristics of patients with TGCT at the Barretos Cancer Hospital.

Characteristics	Patients
	n (%)
TGCT	97
Age	
< 30 years	49 (50.5%)
≥ 30 years	48 (49.5%)
Histological group	
Non-seminoma	56 (57.7%)
Seminoma	41 (42.3%)
Histology	
Pure Non-seminoma	16 (16.5%)
Pure Seminoma	41 (42.3%)
Mixed Tumor	40 (41.2%)
Stages (AJCC)	
IS	7 (7.2%)
I	25 (25.8%)
II	27 (27.8%)
III	38 (39.2%)
Risk (IGCCCG)	
Low (good prognostic)	38 (39.2%)
Intermediate	15 (15.5%)
High (poor prognostic)	12 (12.4%)
N/A	32 (33.0%)
Chemotherapy	
PEB	56 (57.7%)
EP	14 (14.4%
TIP	1 (1.0%)
Ignored	26 (26.8%)
Chemosensitivity	
Responsive	62 (63.9%)
Refractory	6 (6.2%)
Ignored	4 (4.1%)
N/A	25 (25.8%)

AJCC, American Joint Committee on Cancer; IGCCCG, International Germ Cell Cancer Collaborative Group.

N/A, Not applicable.

PEB, Bleomycin, Etoposide and Platinum.

EP, Etoposide and Platinum.

TIP, Paclitaxel, Ifosfamide, and Cisplatin.

The most used treatment strategy was the bleomycin, etoposide, and cisplatin (PEB) combination (57.7%). Considering all of the chemotherapy regimens, 63.9% were responsive and 6.2% were refractory. The remaining 29.9% corresponds to those who did not undergo chemotherapy (n = 25) and/or to those who died (n = 4) before the date set for the first cycle.

The overall survival probability of the analyzed cohort was 91.5% in the 5-year follow-up period and 83.2% in the 10-year follow-up period; the overall survival according to AJCC staging, histological group, IGCCCG risk, and chemosensitivity status were evaluated and did not differ from the results in the literature (**Figure S2**).

### 3.2 CNV analysis for *KRAS*

**Figure 1:**
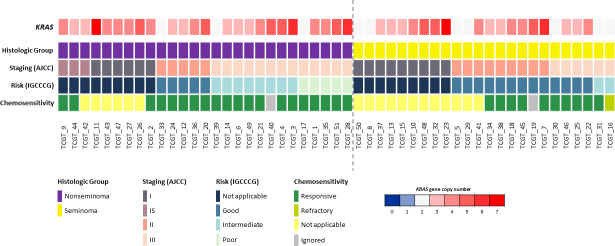
Copy number variations of the *KRAS* gene compared to clinicopathologic data (histological group, staging, risk and chemosensitivity). Only TGCTs evaluated for copy number variation are presented (n = 51). Each column represents an individual patient. The dotted line in the center of the figure separates seminoma and non-seminoma cases. A color scale (blue to red) is used to represent the variation in the number of copies of the *KRAS* gene, in which the dark blue color represents the deletion of two copies of this gene (0 copies); light blue the deletion of only one copy; white represents that there is no loss or gain of copies; and light red to dark red colors represent a gain of copies of this gene (1 to 7 copies).

In total, 51 cases were analyzed for *KRAS* CNV, and it was observed that 41 cases (80.4%) showed at least one copy number gain, with a variation of three to seven *KRAS* copies (**Figure 1**). Non-seminoma cases showed a non significant trend of greater variation in the number of copies of the *KRAS* gene, when compared to seminoma cases (47.1% *vs* 33.3%; p = 0.160), and none of the cases showed loss of copy number.

**Figure 2:**
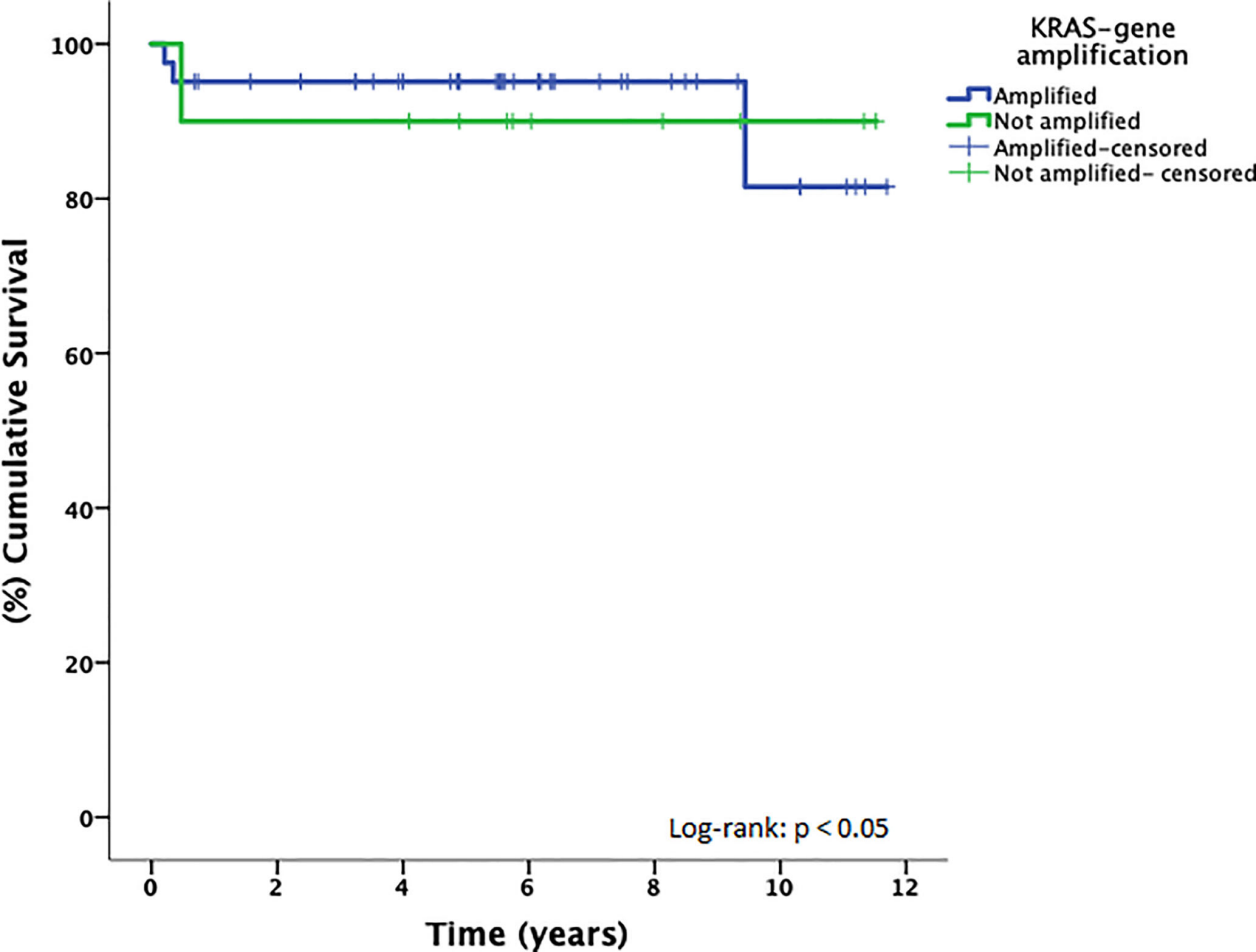
Overall Survival stratified by *KRAS* copy number in TGCT.

*KRAS* copy gain was compared to the clinicopathologicdata of the TGCT patients, including age, histological group, stage (AJCC), risk (IGCCCG), chemosensitivity to cisplatin-based treatment, and overall survival; however, no significant association between *KRAS* CNV status and patient’s features was observed (**Table S1**). Regarding overall survival (10-year period), the *KRAS* copy gain group presented a worse prognosis compared with the group with no *KRAS* copy gain (10y-OS, 90% *vs*. 81.5%, p = 0.048) (**Figure 2**).

### 3.3 Called variants by next generation sequencing

Sixty-five cases were analyzed for the presence of variants and a total of 82,975 variants were initially detected; the variants were filtered out according to the pipeline, and we identified 73 different variants (amongst them, pathogenic, likely pathogenic, VUS, and drug response) (**Table S2**). Furthermore, after CGI was employed to verify the variant prediction status, only known variants and/or predicted drivers in cancer-related genes were maintained. So, among the 65 cases analyzed for the presence of variants in this study, 35 cases (53.8%) showed a total of 51 different variants (known and/or predicted as drivers) that were identified in 11 of 15 genes of the panel.

**Figure 3:**
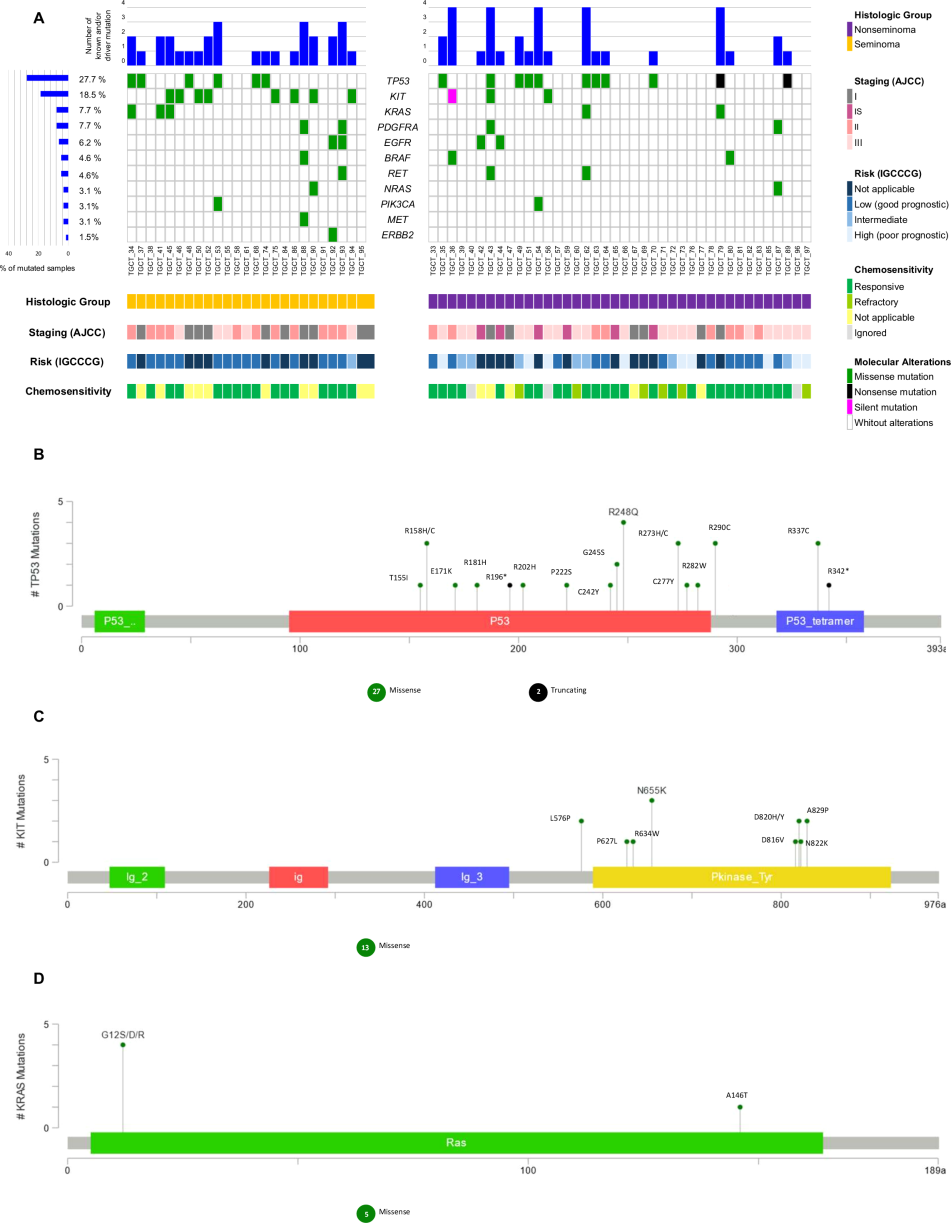
Waterfall plot of the known and/or predicted driver mutation spectrum of Testicular Germ Cell Tumor (TGCT). **(A)** Plots show the frequency of samples mutated for seminomas and non-seminomas. The upper panel demonstrates the frequency of mutation for each sample. The left panel shows the frequency of samples harboring mutations according to the gene. The lower panel indicates clinicopathologic data. **(B)** Variant distribution in TP53 gene. **(C)** Variant distribution in *KIT* gene. **(D)** Variant distribution in *KRAS* gene. All graphs depict a lollipop plot showing identified variants relative to a schematic representation of the gene. Any position with a mutation is shown with a circle, and the length of the line depends on the number of mutations detected at that codon. The grey bar represents the entire protein with different amino acid positions (aa). The colored boxes are specific functional domains. On top of the lollipops, the most frequent variants are annotated as the amino acid change at that specific site.

*TP53* alterations were the most common in our cases (27.7%, n=18), followed by *KIT* (18.5%, n=12), *KRAS* (7.7%, n=5), *PDGFRA* (7.7%, n= 5), *EGFR* (6.2%, n=4), *BRAF* (4.6%, n= 3), *RET* (4.6%, n=3), *NRAS* (3.1%, n= 2), *PIK3CA* (3.1%, n= 2), *MET* (3.1%, n=2) and *ERBB2* (1,5%, n=1). No variants were identified on *AKT1*, *FOXL2*, *GNAQ*, or *GNA11* genes in any of the cases (**Figure 3**).

We further analyzed the variants according to the histologic group, staging (AJCC), risk (IGCCCG), and chemosensitivity (**Figure 3**). The frequency of known and/or predicted driver mutations in seminomas was higher (72% of cases with at least one variant) when compared to non-seminomas (42.5% of cases with at least one variant) (p = 0.024) (**Table S3**).

*TP53*, *KIT*, and *KRAS* genes were the three most mutated in our case series; *TP53*/*KIT* and *KIT*/*KRAS* genes were mutually exclusively mutated, except for a non-seminoma case and a seminoma case, respectively.

Mutations in the *TP53* gene were identified more frequently among non-seminomas when compared to seminomas, and more than one variant was observed for the same case. Three non-seminoma cases and one seminoma case, all of which were chemotherapy-responsive, carried the R248Q variant. The R337C and R290C variants were observed in more than two cases. A case of poor prognostic non-seminoma presented three distinct variants, and another four cases also presented two or more variants concomitantly (**Figures 3A, B**; **Table S2**).

Only one chemotherapy-refractory non-seminoma case (staging III and intermediate risk) presented two different variants in the *TP53* gene (R158C and C277Y) (**Table S2**).

Mutations in the *KIT* gene were identified more frequently among seminomas when compared to non-seminomas, and more than one variant was observed in only one case. L576P and A829P variants were observed in more than one case. One non-seminoma case with a poor prognosis, a carrier of the N655K variant, died before the date set for the first cycle of adjuvant chemotherapy, at 20 years old. No variant in the *KIT* gene was found in chemotherapy-refractory cases (**Figures 3A–C**; **Table S2**).

Mutations in the *KRAS* gene were identified more frequently among seminomas when compared to non-seminomas, and four different variants were observed in five TGCT cases. One non-seminoma case and one seminoma case, both chemotherapy-responsive, carried the G12S variant. Two other variants, also on the *KRAS* gene codon 12 (G12R and G12D), were observed in seminoma cases. The A146T variant was identified in non-seminoma case (staging III and intermediate risk) (**Figures 3A–D**; **Table S2**).

Analysis comparing the mutational status of genes with clinicopathologic data revealed that *KIT* (p = 0.007) was significantly mutated in seminomas, and *EGFR* (p = 0.010) in stage IS. All clinical and molecular comparisons of the other genes are shown in **Table S4**.

We further compared the mutational status with overall survival, but no significant association was found (**Tables S5, 6**; **Figure S3**).

## 4 Discussion

The analysis of tumor mutation and *KRAS* copy number variation revealed expressive *KRAS* copy number gain, well-known variants and potentially targetable variants never described before in TGCT.

The findings of *KRAS* copy number gain might be explained due to the gain of the 12p isochromosome, commonly found in almost all TGCTs, especially in non-seminomas. *KRAS* is located in the 12p11.2–p12.1 region of chromosome 12, and its overexpression seems to be associated with the development of TGCTs (17, 37). In seminomas, Loveday et al. (17) demonstrated that *KRAS* copy number was relatively high, but Shen et al. (18) identified a subset of seminomas with decreased *KRAS* copy number. We were not able to identify statistical differences between seminomas and non-seminomas regarding *KRAS* copy number gain, and none of our cases had loss of copy number. Therefore, the role of the *KRAS* copy number in TGCT needs to be explored further. Worse cancer-specific and overall survival have been associated to *KRAS* mutation and copy number gain in lung cancer (38, 39). Using cell free DNA somatic mutation analysis, *KRAS* mutation and copy number gain were associated to worse outcomes in pancreatic cancer (40). We demonstrated a worse prognosis associated to *KRAS* copy gain when compared to the group with no gain, and this finding has not been addressed before in TGCT.

The gain of 12-p occurs *in utero* at the gonocyte stage and the development of GCNIS, a TGCT precursor, after birth (41). This pathogenesis involves the blood–testis barrier, which harbors a specialized microenvironment and an immunological barrier that prevents the production of antibodies against meiotic and postmeiotic germ cells (42). This complex and overprotected microenvironment might, at least in part, explain why point mutations are rare events in TGCTs. Although rare, mutation might occur mainly in the *TP53*, *KIT*, *KRAS*, *BRAF*, and *NRAS* genes (18, 41, 43).

*TP53* is one of the most frequently mutated genes in different types of cancer (44, 45) and variants in *TP53* have been identified in non-seminomas with no changes in seminomas (46). However, the COSMIC database shows that mutations in *TP53* are rare in TGCTs, with a frequency of 4% considering all histological subtypes. Here, among the known and predicted driver mutations, 26% were found in the *TP53* gene, which was the most commonly mutated in our series of cases, mainly in non-seminomas. Fifteen variants were detected in *TP53*, and ten of them (R290C, R158H, G245S, C242Y, C277Y, E171K, R158C, R181H, R196* and R342*) have not been described in TGCT previously. Although there were no cases of Li–Fraumeni syndrome in our dataset, some of the variants found were associated with that syndrome (R282W, R342*, R196*) in some aspects (47–50). *TP53* has been associated to the platinum-resistance mechanism (51), but we did not find any association, and there is no evident cause for the high frequency of *TP53* in our study.

*KIT* mutation is more prevalent in seminomas, and it was the second most frequently mutated gene among our cases, with almost all cases being seminomas (23, 25, 52, 53). *KIT* variants have been identified in TGCT precursor cells, suggesting that the *KIT* gene may be largely responsible for the development of TGCTs, and the identification of these variants might assist in the diagnosis of the disease at an early stage (54). We found eight variants of *KIT*, and it is noteworthy that the D816V, D820H, and D820Y variants, located in the same region as other *KIT* mutations (exon 17), presumably also encode a constitutively activated protein that confers resistance to imatinib (55, 56). Addionally, *KRAS* mutations have been associated to cisplatin-resistant TGCTs (57), but we did not find any association in our results. We found four *KRAS* variants (A146T, G12D, G12R, G12S) and two *NRAS* variants (G12A, G60R), and this is the first time the *NRAS* G60R variant has been described. None of the *PDGFRA* variants identified have been previously described in TGCT either.

Interestingly, targetable variants were found in genes such as *BRAF*, *EGFR*, and *RET*. *BRAF* mutations are rare in TGCT (28, 52) and the majority of anti-BRAF drugs target V600E variants. However, we found three cases of *BRAF* T599I variants, and it has been suggested that vemurafenib might control cancer, such as melanoma, even in patients with that variant (58). Four genomic alterations were detected in *EGFR* (A822T, R776H, S768N, T790M), and the T790M variant has been described in advanced non-small cell lung cancer (NSCLC) as an acquired variant associated with Tyrosine Kinase Inhibitors (TKIs) resistance (59, 60). A third generation of anti-EGFR drugs has been developed to overcome this resistance in lung cancer with the T790M variant, including osimertinib (61). *RET* inhibitors such as pralsetinib and selpercatinib have been developed and approved by the FDA to treat patients with *RET* alterations (62). We found two different variants in *RET* (R912W, T930M), and none of them have been reported in TGCT before. Although rare, targetable mutations in TGCT increase the opportunities for precision medicine within the scenario of the accelerated approval of new drugs, mainly in cases of refractory disease.

There are limitations to our study, such as the low number of TGCT cases, the retrospective design, the single institution setting, the low number of events to analyze survival, and the large number of mixed tumors. All these limitations increase bias and might lead to misinterpretation of the results. Additionally, the sequencing analysis of the presence study was limited tumor tissue, since it was a retrospective nature of the study and we did not have availability of blood or normal tissue of the cases. Therefore, we could not exclude the germline or somatic nature of the variants identified. The analysis based on important and consolidated databases might minimize the presence of polymorphisms. However, we found new variants never described before in TGCT, and we were able to show targetable variants using a reliable mutational panel. Moreover, patients with admixed American ancestry, like Brazilian people, are commonly underrepresented in genomic profile studies, although it has been increasing (63). The populations previously studied are mostly European, so the cases in our Brazilian cohort are expressive, and might bring new insights and generate new hypotheses in precision oncology.

In conclusion, the increase in the number of copies of the *KRAS* gene is a very frequent event in TGCT, and non-seminomas are most highly associated with a higher rate of this amplification. Although point mutations are rare events in TGCTs, relevant gene variants are identified mainly in *TP53*, *KIT*, *KRAS*, and *NRAS* genes, and 40% of the evaluable genes have at least one known and/or predicted driver variant in one of the 15 genes tested, with *TP53* being the most commonly mutated gene. Although larger studies enrolling collaborative networks might shed light on the molecular landscape of TGCTas well as limited evidence available for use of targeted approaches in TGCT, our findings unveil the potential of actionable variants in clinical management when applying targeted therapies, and further evaluation of these approaches is necessary.

## Data availability statement

The data presented in the study are deposited in the Sequence Read Archive (SRA) repository, accession number PRJNA942937 (https://www.ncbi.nlm.nih.gov/sra/PRJNA942937)

## Ethics statement

The studies involving human participants were reviewed andapproved by Barretos Cancer Hospital IRB under Protocol No. 784/2014. Written informed consent for participation was not required for this study in accordance with the national legislation and the institutional requirements.

## Author contributions

FC, MTP, and RR designed the study and critically revised the results. EC and MFP performed the study. AL, MR, and LL supervised the experiments. EC, ML, AS and IP analyzed the data. EC wrote the manuscript draft. FC, MTP, RR, LL, and MR revised the manuscript. All authors contributed to the article and approved the submitted version
