# Supplementary material for: Somatic mutation detection and KRAS amplification in testicular germ cell tumors
Source: Front Oncol. 2023 Mar 16;13:1133363. doi: 10.3389/fonc.2023.1133363 (PMC10060882; doi:10.3389/fonc.2023.1133363)
Supplement: Supplementary file 1 [file DataSheet_1.zip › Figure S2.PDF]

Figure S2 - Overall survival according to AJCC staging, histological group, IGCCCG risk, and chemosensitivity status.

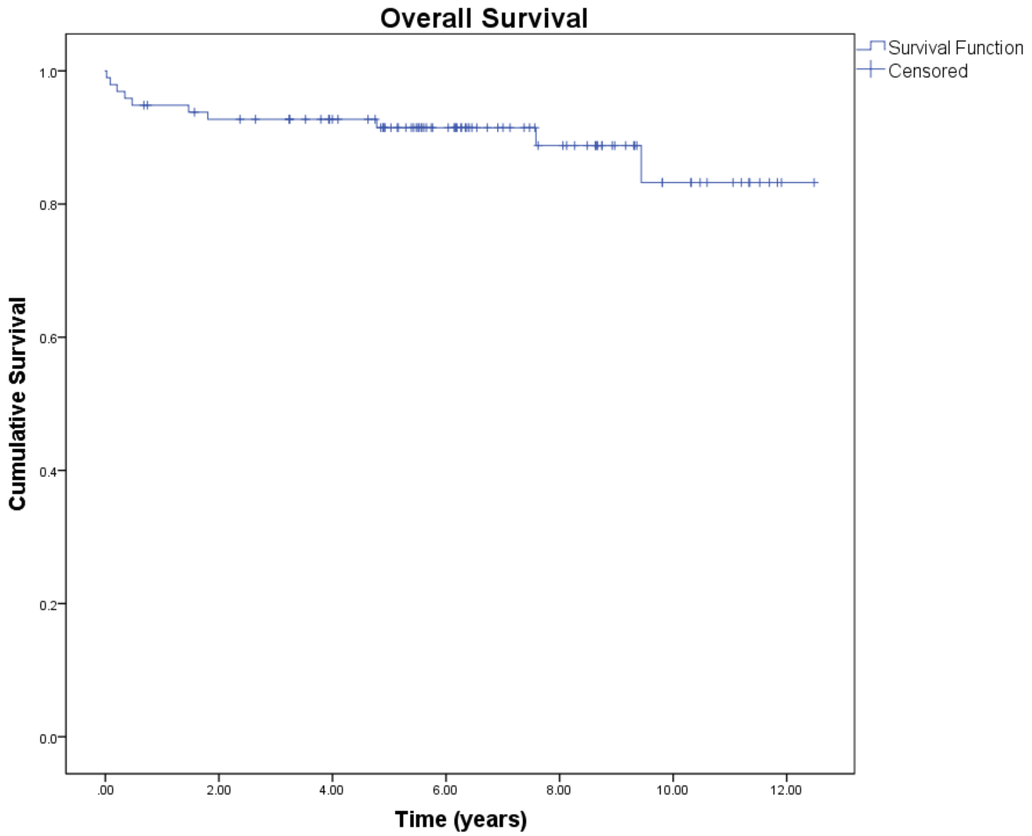

Year	2	4	6	8	10	12
**Overall Survival**						
At Risk	90	90	89	88	87	87

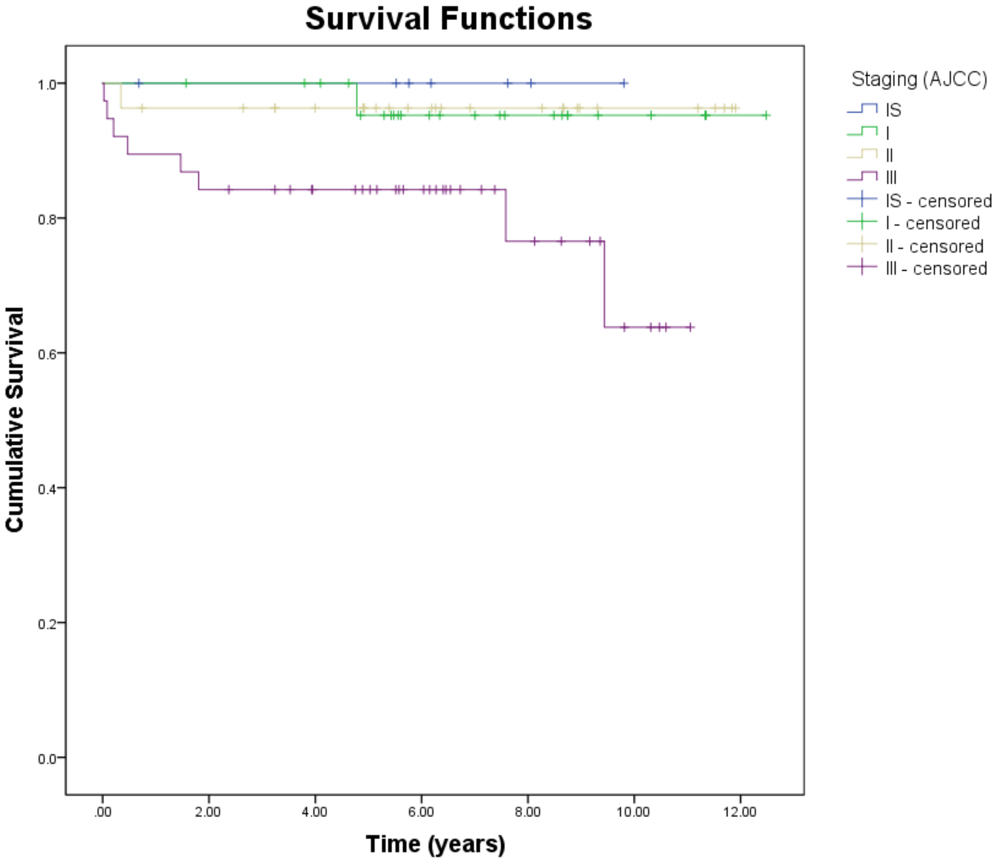

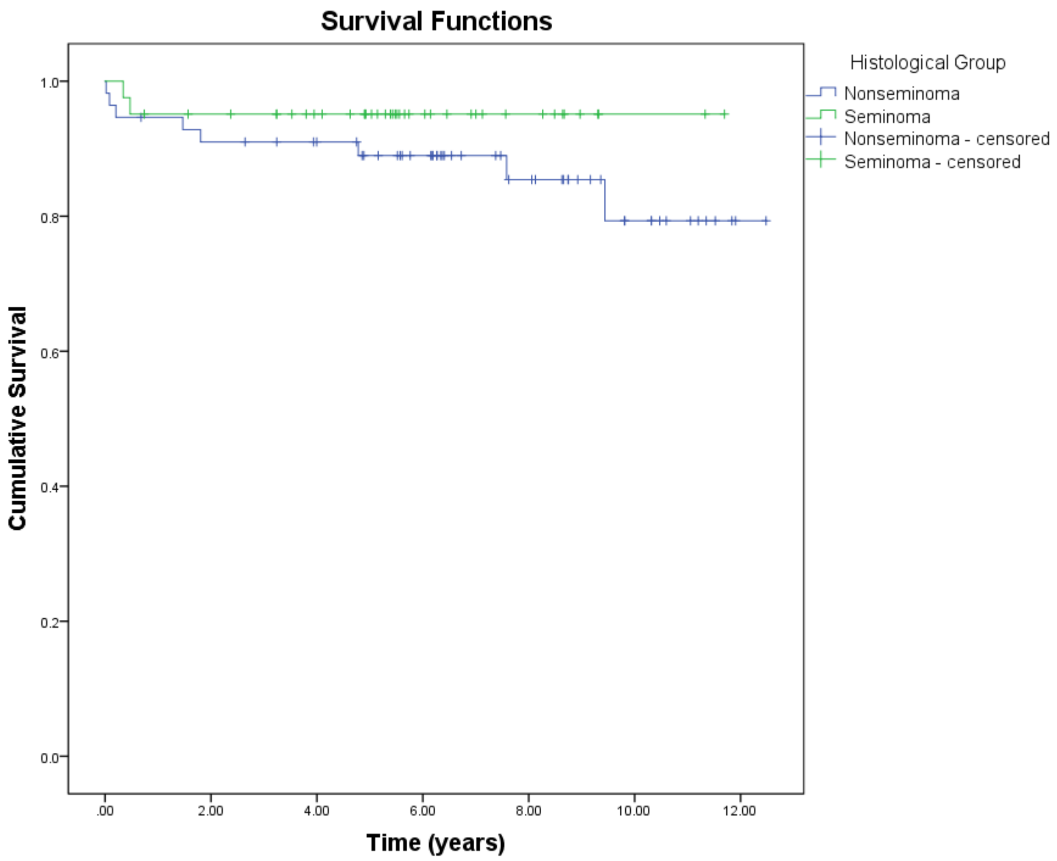

**Year**	**2**	**4**	**6**	**8**	**10**	**12**
**Staging IS**						
At Risk	6	6	6	6	-	-
**Staging I**						
At Risk	25	25	24	24	24	24
**Staging II**						
At Risk	26	26	26	26	26	26
**Staging III**						
At Risk	32	32	32	31	30	-

**Year**	**2**	**4**	**6**	**8**	**10**	**12**
**Nonseminoma**						
At Risk	51	51	50	49	48	48
**Seminoma**						
At Risk	39	39	39	39	39	-

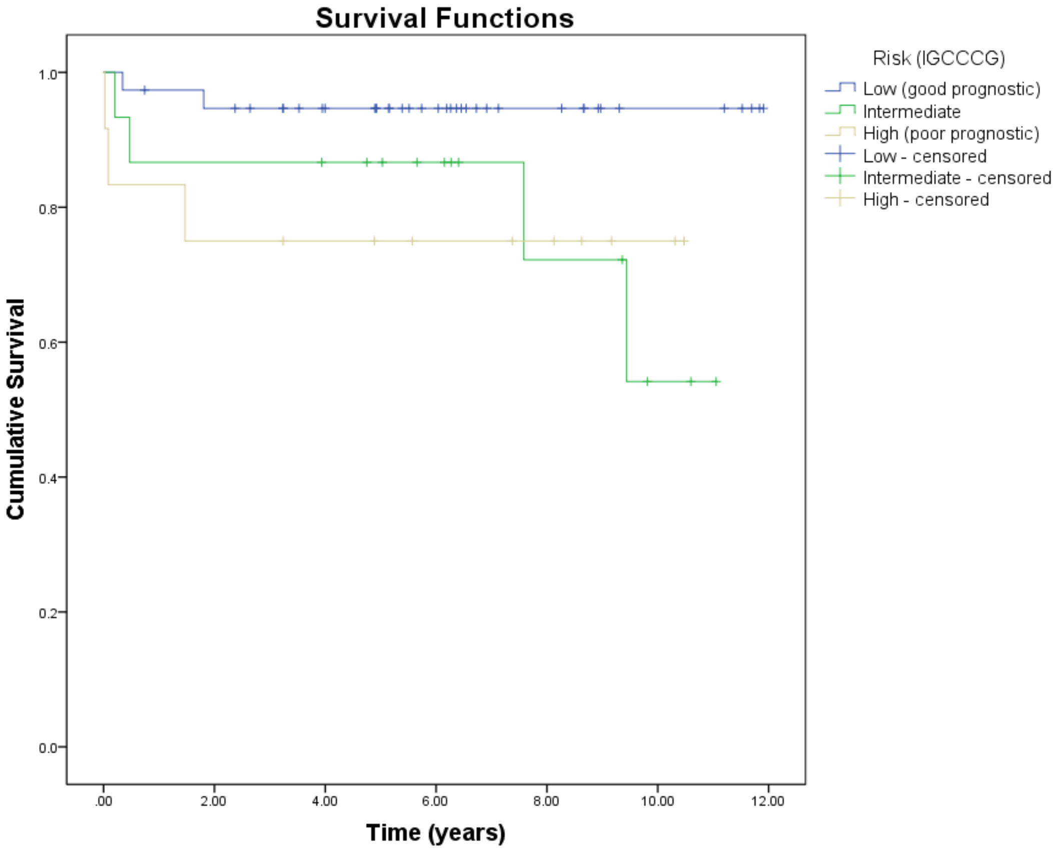

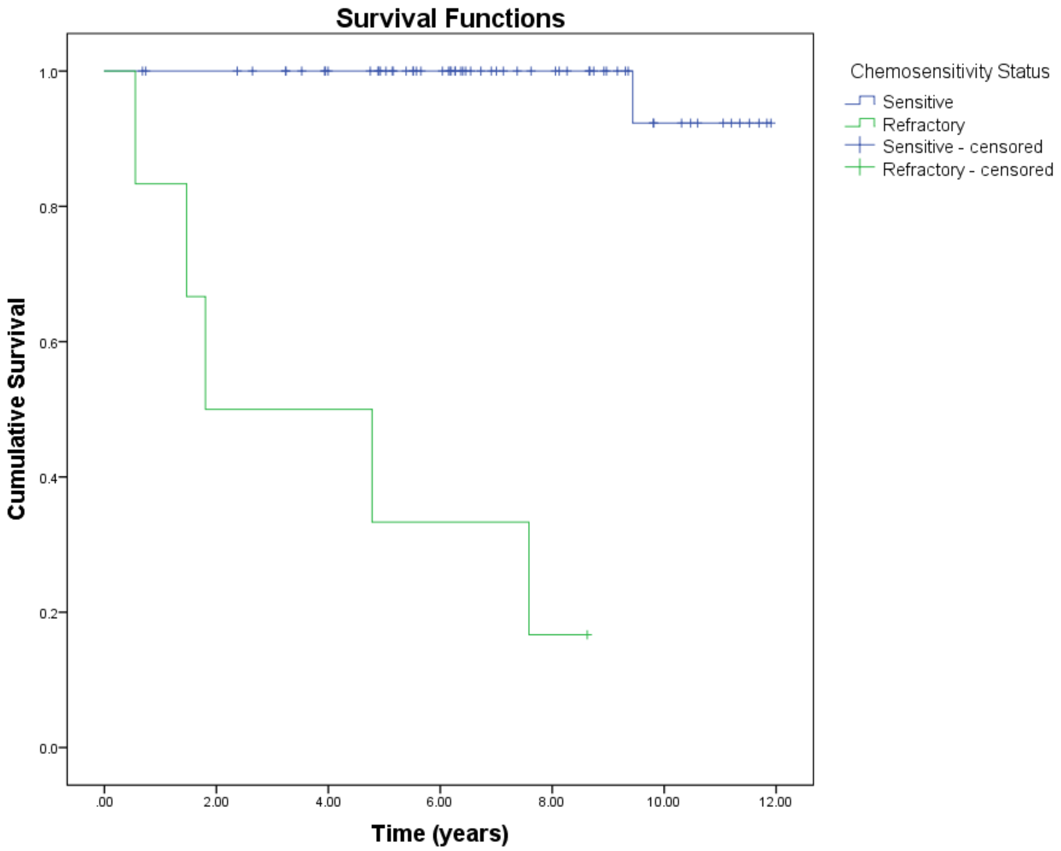

**Year**	**2**	**4**	**6**	**8**	**10**	**12**
**Low (good)**						
At Risk	36	36	36	36	36	36
**Intermediate**						
At Risk	13	13	13	12	11	-
**High (poor)**						
At Risk	9	9	9	9	9	-

**Year**	**2**	**4**	**6**	**8**	**10**	**12**
**Sensitive**						
At Risk	62	62	62	62	61	61
**Refractory**						
At Risk	3	3	2	1	-	-
