# Supplementary material for: Somatic mutation detection and KRAS amplification in testicular germ cell tumors
Source: Front Oncol. 2023 Mar 16;13:1133363. doi: 10.3389/fonc.2023.1133363 (PMC10060882; doi:10.3389/fonc.2023.1133363)
Supplement: Supplementary file 1 [file DataSheet_1.zip › Table S1.DOCX]

| **Table S1** – Comparison of clinicopathological features of TGCT patients with *KRAS* copy number. | | | | | |
| --- | --- | --- | --- | --- | --- |
| **Variables** | **Parameters** | **n** | ***KRAS* copy number** | | |
|  |  |  | **YES (%)** | **NO (%)** | **p-value** |
| **Age** | < 30 years | 25 | 21 (84.0) | 4 (16.0) | 0.726 |
|  | ≥ 30 years | 26 | 20 (76.9) | 6 (23.1) |  |
|  |  |  |  |  |  |
| **Histological group** | Non-seminoma | 27 | 24 (47.1) | 3 (5.9) | 0.160 |
|  | Seminoma | 24 | 17 (33.3) | 7 (13.7) |  |
|  |  |  |  |  |  |
| **Stages (AJCC)** | IS | 3 | 3 (5.9) | 0 (0.0) | 0.861 |
|  | I | 15 | 13 (25.5) | 2 (3.9) |  |
|  | II | 14 | 11 (21.6) | 3 (5.9) |  |
|  | III | 19 | 14 (27.5) | 5 (9.8) |  |
|  |  |  |  |  |  |
| **Risk (IGCCCG)** | Low (good prognostic) | 18 | 14 (42.5) | 4 (12.1) | 0.861 |
|  | Intermediate | 10 | 7 (21.2) | 3 (9.1) |  |
|  | High (poor prognostic) | 5 | 4 (12.1) | 1 (3.0) |  |
|  |  |  |  |  |  |
| **Chemosensitivity** | Responsive | 30 | 25 (80.7) | 5 (16.1) | 0.194 |
|  | Refractory | 1 | 0 (0.0) | 1 (3.2) |  |

AJCC: American Joint Committee on Cancer;

IGCCCG: *International Germ Cell Cancer Collaborative Group*
