# Supplementary material for: Somatic mutation detection and KRAS amplification in testicular germ cell tumors
Source: Front Oncol. 2023 Mar 16;13:1133363. doi: 10.3389/fonc.2023.1133363 (PMC10060882; doi:10.3389/fonc.2023.1133363)
Supplement: Supplementary file 1 [file DataSheet_1.zip › Table S3.DOCX]

**Table S3** - Comparison of clinicopathological features of TGCT patients with frequency of known and/or predicted driver mutation.

| **Variables** | **Parameters** | **n** | **Known and/or predicted driver mutation** | **WT** | **p-value** |
| --- | --- | --- | --- | --- | --- |
|  |  |  |  |  |  |
| **Histologic Group** | Seminoma | 25 | 18 (72.0%) | 7 (28.0%) | **0.024** |
|  | Nonseminoma | 40 | 17 (42.5%) | 23 (57.5%) |  |
| **Staging (AJCC)** | IS | 6 | 4 (66.7%) | 2 (33.3%) | 0.189 |
|  | I | 15 | 11 (73.3%) | 4 (26.7%) |  |
|  | II | 19 | 12 (63.2%) | 7 (36.8%) |  |
|  | III | 25 | 10 (40.0%) | 15 (60.0%) |  |
| **Risk (IGCCCG)** | Low (Good prognostic) | 27 | 15 (55.6%) | 12 (44.4%) | 0.581 |
|  | Intermediate | 8 | 4 (50.0%) | 4 (50.0%) |  |
|  | High (Poor prognostic) | 9 | 3 (33.3%) | 6 (66.7%) |  |
| **Chemosensitivity** | Responsive | 42 | 24 (57.1%) | 18 (42.9%) | 0.077 |
|  | Refractory | 6 | 1 (16.7%) | 5 (83.3%) |  |

AJCC: American Joint Committee on Cancer

IGCCCG: International Germ Cell Cancer Collaborative Group

WT: Wild type
