# Supplementary material for: Somatic mutation detection and KRAS amplification in testicular germ cell tumors
Source: Front Oncol. 2023 Mar 16;13:1133363. doi: 10.3389/fonc.2023.1133363 (PMC10060882; doi:10.3389/fonc.2023.1133363)
Supplement: Supplementary file 1 [file DataSheet_1.zip › Table S5.DOCX]

**Table S5** – Comparison of overall survival of TGCT patients with mutational status of genes.

| **Genes** | **Parameters** | **n** | **Overall Survival**  **(2 years) %** | **Overall Survival**  **(5 years) %** | **p-value** |
| --- | --- | --- | --- | --- | --- |
| ***KIT*** | **Mutated** | 12 | 96.3 | 96.3 | 0.148 |
|  | **WT** | 53 | 89.3 | 86.2 |  |
| ***TP53*** | **Mutated** | 18 | 96.2 | 91.8 | 0.924 |
|  | **WT** | 47 | 89.6 | 89.6 |  |
| ***PDGFRA*** | **Mutated** | 5 | 100 | 91.7 | 0.673 |
|  | **WT** | 60 | 90.2 | 90.2 |  |
| ***KRAS*** | **Mutated** | 5 | 100 | 100 | 0.244 |
|  | **WT** | 60 | 90.9 | 88.7 |  |
| ***NRAS*** | **Mutated** | 2 | 100 | 100 | 0.412 |
|  | **WT** | 63 | 91.4 | 89.3 |  |
| ***EGFR*** | **Mutated** | 4 | 100 | 100 | 0.420 |
|  | **WT** | 61 | 91.5 | 89.4 |  |
| ***PIK3CA*** | **Mutated** | 2 | 100 | 100 | 0.536 |
|  | **WT** | 63 | 91.8 | 89.9 |  |
| ***BRAF*** | **Mutated** | 3 | 100 | 100 | 0.524 |
|  | **WT** | 62 | 91.8 | 89.8 |  |
| ***RET*** | **Mutated** | 3 | 100 | 100 | 0.548 |
|  | **WT** | 62 | 91.8 | 89.8 |  |
| ***MET*** | **Mutated** | 2 | 100 | 80.0 | 0.525 |
|  | **WT** | 63 | 91.5 | 91.5 |  |
| ***ERBB2*** | **Mutated** | 1 | 100 | 100 | 0.749 |
|  | **WT** | 64 | 92.1 | 90.2 |  |

WT: wild‐type
