# Supplementary material for: Somatic mutation detection and KRAS amplification in testicular germ cell tumors
Source: Front Oncol. 2023 Mar 16;13:1133363. doi: 10.3389/fonc.2023.1133363 (PMC10060882; doi:10.3389/fonc.2023.1133363)
Supplement: Supplementary file 1 [file DataSheet_1.zip › Table S6.DOCX]

**Table S6** – Comparison of overall survival of stages IS, II and III TGCT patients with genetic mutation status.

| **Genes** | **Parameters** | **n** | **Overall Survival**  **(2 years) %** | **Overall Survival**  **(5 years) %** | **p-value** |
| --- | --- | --- | --- | --- | --- |
| ***KIT*** | **Mutated** | 20 | 95.0 | 95.0 | 0.222 |
|  | **WT** | 30 | 86.4 | 86.4 |  |
| ***TP53*** | **Mutated** | 19 | 94.7 | 94.7 | 0.608 |
|  | **WT** | 31 | 86.8 | 86.8 |  |
| ***PDGFRA*** | **Mutated** | 7 | 100.0 | 100.0 | 0.308 |
|  | **WT** | 43 | 88.1 | 88.1 |  |
| ***KRAS*** | **Mutated** | 7 | 100.0 | 100.0 | 0.292 |
|  | **WT** | 43 | 88.1 | 88.1 |  |
| ***NRAS*** | **Mutated** | 43 | 100.0 | 100.0 | 0.560 |
|  | **WT** | 7 | 89.2 | 89.2 |  |
| ***EGFR*** | **Mutated** | 5 | 100.0 | 100.0 | 0.443 |
|  | **WT** | 45 | 88.8 | 88.8 |  |
| ***PIK3CA*** | **Mutated** | 3 | 100 | 100 | 0.504 |
|  | **WT** | 47 | 89.2 | 89.2 |  |
| ***BRAF*** | **Mutated** | 2 | 100.0 | 100.0 | 0.568 |
|  | **WT** | 48 | 89.4 | 89.4 |  |
| ***RET*** | **Mutated** | 2 | 100.0 | 100.0 | 0.602 |
|  | **WT** | 48 | 89.4 | 89.4 |  |
| ***MET*** | **Mutated** | 2 | 100.0 | 100.0 | 0.602 |
|  | **WT** | 48 | 89.4 | 89.4 |  |
| ***ERBB2*** | **Mutated** | 1 | 100.0 | 100.0 | 0.742 |
|  | **WT** | 49 | 89.6 | 89.6 |  |

WT: wild‐type
