# Supplementary figures and images for: Somatic mutation detection and KRAS amplification in testicular germ cell tumors
Source: Front Oncol. 2023 Mar 16;13:1133363. doi: 10.3389/fonc.2023.1133363 (PMC10060882; doi:10.3389/fonc.2023.1133363)

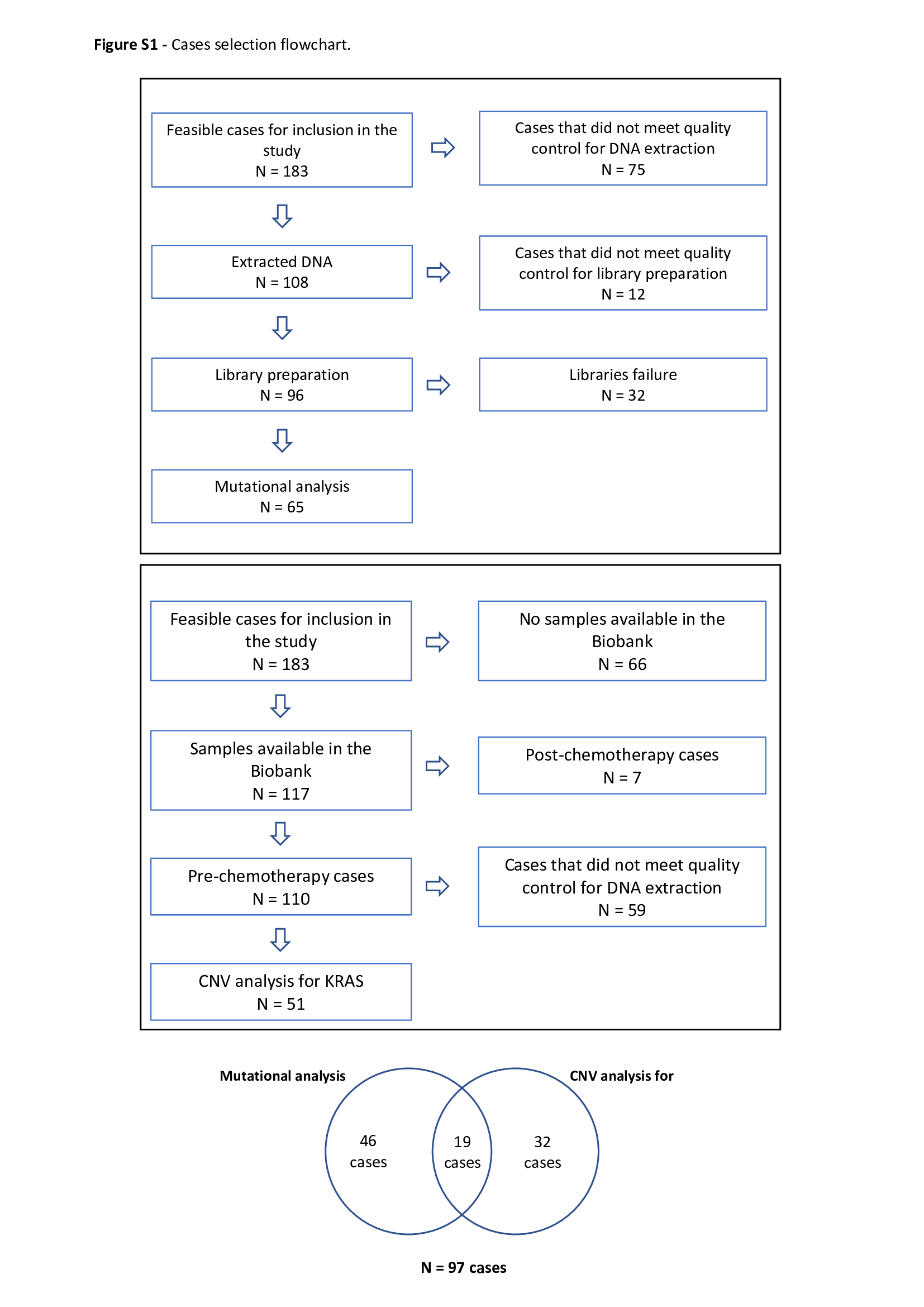

Supplement: Supplementary file 1 [file DataSheet_1.zip › Figure S1.PNG]

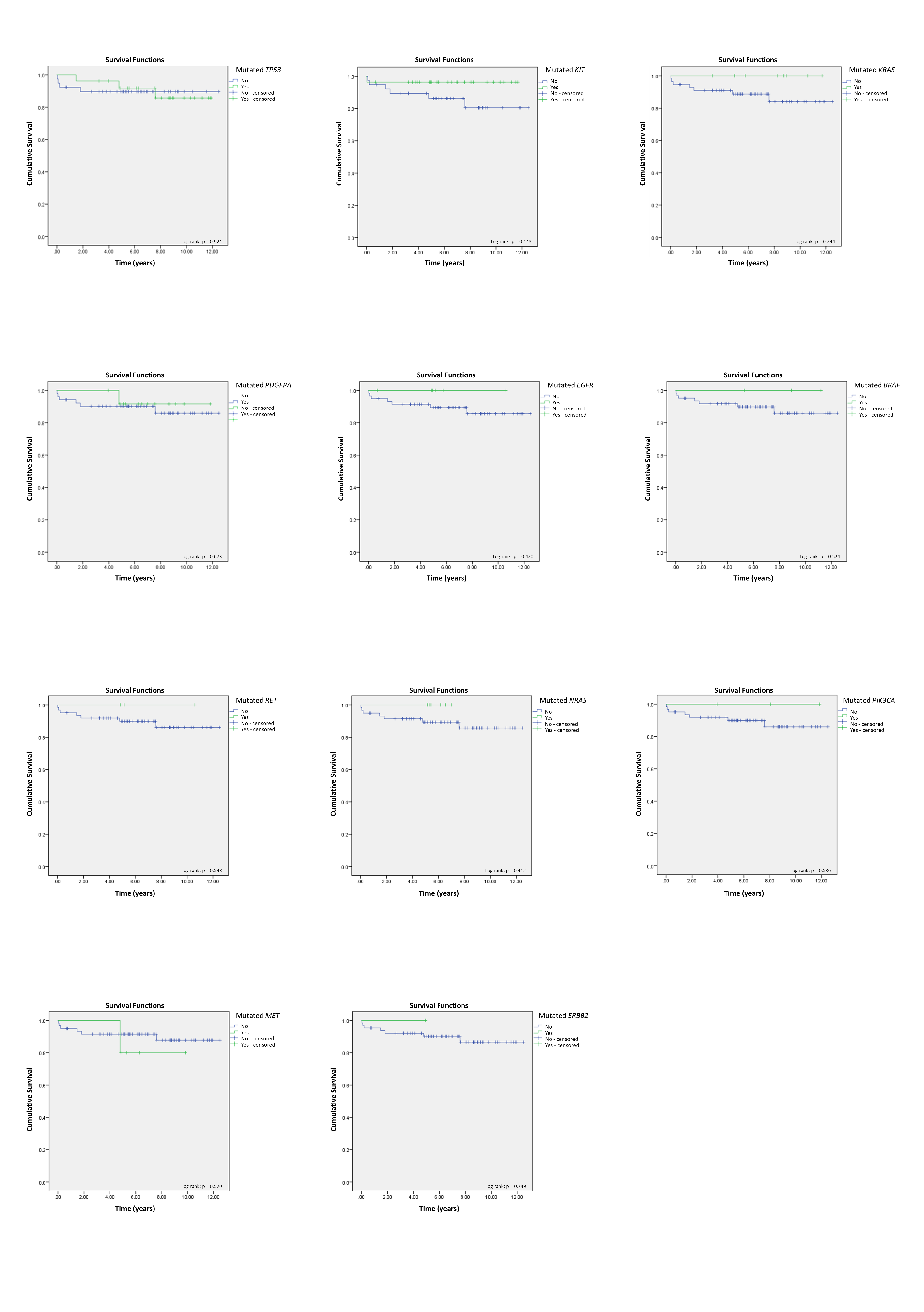

Supplement: Supplementary file 1 [file DataSheet_1.zip › Figure S3.TIF]
